# Cfp1 is required for gene expression-dependent H3K4 trimethylation and H3K9 acetylation in embryonic stem cells

**DOI:** 10.1186/s13059-014-0451-x

**Published:** 2014-09-04

**Authors:** Thomas Clouaire, Shaun Webb, Adrian Bird

**Affiliations:** Wellcome Trust Centre for Cell Biology, University of Edinburgh, Edinburgh, EH9 3JR UK; Current address: LBCMCP, Université Paul Sabatier- CNRS UMR 5088, 118 Route de Narbonne, 31062 Toulouse, Cedex France

## Abstract

**Background:**

Trimethylation of histone H3 lysine 4 (H3K4me3) accumulates at promoters in a gene activity-dependent manner. The Set1 complex is responsible for most H3K4me3 in somatic cells and contains the conserved subunit Cfp1, which is implicated in targeting the Set1 complex to CpG islands in mammals. In mouse embryonic stem cells, Cfp1 is necessary for H3K4me3 accumulation at constitutively active gene promoters, but is not required to maintain steady-state transcription of the associated gene.

**Results:**

Here we show that Cfp1 is instrumental for targeting H3K4me3 to promoters upon rapid transcriptional induction in response to external stimuli. Surprisingly, H3K4me3 accumulation is not required to ensure appropriate transcriptional output but rather plays gene-specific roles. We also show that Cfp1-dependent H3K4me3 deposition contributes to H3K9 acetylation genome-wide, suggesting that Cfp1-dependent H3K4me3 regulates overall H3K9 acetylation dynamics and is necessary for histone acetyl transferase recruitment. Finally, we observe increased antisense transcription at the start and end of genes that require Cfp1 for accurate deposition of H3K4me3 and H3K9ac.

**Conclusions:**

Our results assign a key role for Cfp1 in establishing a complex active promoter chromatin state and shed light on how chromatin signaling pathways provide context-dependent transcriptional outcomes.

**Electronic supplementary material:**

The online version of this article (doi:10.1186/s13059-014-0451-x) contains supplementary material, which is available to authorized users.

## Background

In eukaryotes, specialized chromatin structures contribute to multiple DNA-related processes, including transcription, replication and repair. Combinations of specific histone post-translational modifications correlate well with the functional status of the underlying DNA sequence - for example, at sites of transcriptional initiation, elongation or at distal regulatory elements [1-4]. Transitions between chromatin states accompany differentiation, cellular reprogramming, and disease processes [2,4]. However, it is unclear whether histone modification patterns are set up as a consequence of ongoing dynamic processes such as transcription or if they perform instructive roles. It is therefore crucial to systematically address the role of individual histone modifications in different contexts. As chromatin marks usually arise in reproducible groups comprising the same set of modifications, it is important to decipher their interdependence to help determine the biological significance of complex, potentially redundant, chromatin states.

H3K4me3 is a mark associated with eukaryotic gene promoters. In yeast, it is a feature of actively expressed genes [5,6], suggesting that it positively influences transcription. In mammals, H3K4me3 is found at active and inactive promoters at a level dependent on gene activity [7,8]. Most promoters in mouse and human are associated with CpG islands (CGIs), which are DNA elements showing high G + C and CpG content that are usually free of DNA methylation [9,10]. CGIs possess a characteristic chromatin structure thought to predispose them towards promoter activity [9,11]. For example, CGIs can directly recruit H3K4me3, favoring transcriptional competence [12]. In mammalian stem cells, H3K4me3 is found together with H3K27me3 at bivalently marked CGI promoters [13,14], which are poised for activation by developmental signals upon lineage commitment.

H3K4 methylation is achieved by conserved enzymatic complexes related to the yeast COMPASS (Complex associated with Set1) [15,16]. Mammalian COMPASS complexes vary in their catalytic component (Setd1A and Setd1B, Mll1 to Mll4) as well as in specific subunits that contribute to their functional diversity (reviewed in [17]). Set1-containing COMPASS is the main H3K4 histone methyltransferase in most organisms [18-21]. Mll1/Mll2 COMPASS-like have gene-specific roles in H3K4me3 deposition [22-24], while Mll3/Mll4 COMPASS-like complexes mainly contribute to H3K4me1 at enhancers [25,26].

CxxC finger protein 1 (Cfp1, CXXC1 or CGBP) is a specific component of Set1-containing complexes [17,27]. Cfp1 binds unmethylated CpGs and targets Set1 and H3K4me3 to most CGIs in somatic cells, regardless of their transcriptional activity [12]. In embryonic stem cells, Cfp1 plays a fundamental role in genome-wide H3K4me3 organization [28]. It is required for strong H3K4me3 enrichment at constitutively active gene promoters, but plays little role in depositing this mark at poised genes, including bivalent promoters [28]. Surprisingly, in stem cells, reduced H3K4me3 deposition at active promoters does not drastically affect steady-state transcription [28,29]. On the other hand, loss of the *Cfp1* gene in mice results in early embryonic lethality [30] and Cfp1-deficiency in somatic cell lines is toxic [12,31]. Thus, it is possible that Cfp1-deficiency impairs the proper induction of transcription programs in response to differentiation signals or to external stimuli like stress, potentially explaining why *Cfp1*^*−/−*^ embryonic stem (ES) cells are unable to differentiate *in vitro* [32].

In this study, we ask how Cfp1 affects H3K4me3 dynamics in rapid, regulated gene expression, using the transcriptional response to DNA damage as a model. We show that in addition to its role in regulating steady-state H3K4me3 deposition in ES cells, Cfp1 is instrumental in targeting this modification to gene promoters upon rapid transcriptional induction. We also observe that the Cfp1-dependent H3K4me3 accumulation that follows gene induction is not strictly required to ensure appropriate transcriptional output but rather plays gene-specific roles. We also identify a strong co-dependency between H3K4me3 and H3K9ac deposition upon transcriptional induction as well as in normally cycling ES cells. Our results suggest that Cfp1-dependent H3K4me3 regulates overall H3K9 acetylation dynamics and is necessary for histone acetyltransferase (HAT) recruitment. Finally, we describe elevated antisense transcription at the start and end of those genes that require Cfp1 for accurate H3K4me3 and H3K9ac deposition.

## Results

### Cfp1 is required for H3K4me3 deposition following transcriptional induction

**Figure 1:**
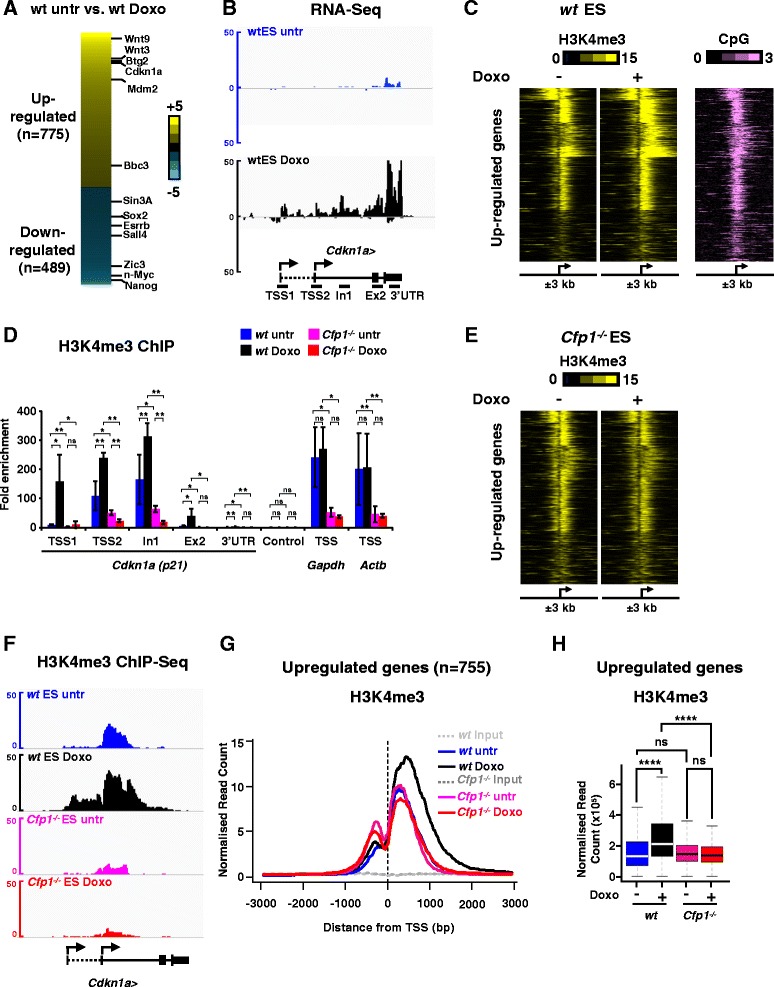
**Cfp1 is crucial for H3K4me3 deposition at promoters of genes regulated upon DNA damage. (A)** Heatmap representing gene expression changes induced by doxorubicin treatment (*P*-value <0.05, fold change ≥ 2.5). Genes highlighted were identified in previous studies [33,34]. Untr, untreated. **(B)** Genome browser screenshots representing RNA-Seq normalized read count in WT ES cells at the *Cdkn1a* locus. Positions for quantitative PCR (qPCR) primers used during the study are also represented. TSS, transcription start site. **(C)** Heatmap showing H3K4me3 normalized read count 3 kb upstream and downstream of the TSS of genes induced by doxorubicin in WT ES cells (n = 755). Unmethylated CpGs are represented by CAP-Seq enrichment in WT ES cells [28]. **(D)** ChIP-qPCR analysis of H3K4me3 enrichment at the *Cdkn1a* locus for untreated or doxorubicin-treated WT and *Cfp1* ^*−/−*^ ES cells. Results are expressed as fold enrichment relative to input DNA and a control intergenic region on chromosome 15. Control corresponds to an intergenic region on chromosome 5. *Gapdh* and *Actb* promoters are also shown. Data are represented as mean ± standard deviation, n = 3. *P*-values were calculated using two-tailed Student’s *t*-tests: *P < 0.05, **P < 0.01; *P* > 0.05 is not significant (ns). **(E)** Same as (C) for *Cfp1* ^*−/−*^ ES cells. **(F)** Genome browser screenshots representing H3K4me3 ChIP-Seq normalized read count in WT (untreated in blue, treated in black) and *Cfp1* ^*−/−*^ (untreated in pink, treated in red) ES cells at the *Cdkn1a* locus. **(G)** Average profile showing H3K4me3 normalized read count at TSSs of genes induced by doxorubicin treatment in WT ES cells. Input DNA is shown as a control. Signal is displayed from −3 kb to +3 kb surrounding each annotated TSS. **(H)** Comparison of H3K4me3 normalized read density at TSSs of upregulated genes, from 3 kb upstream to 3 kb downstream. *P*-values were calculated using two-tailed unpaired Wilcoxon tests: *P < 0.05, **P < 0.01, ***P < 0.001, ****P < 0.0001.

To determine the role of Cfp1 in H3K4me3 deposition upon rapid transcriptional activation, we analyzed the transcriptional response to doxorubicin, a DNA damaging agent that triggers sudden p53-dependent gene expression changes in mouse ES cells [29,33,34]. Doxorubicin treatment (1 μM, 6 hours) in wild-type (WT ) ES cells rapidly caused increased binding of phosphorylated p53 (at serine 18 in mouse) at the *Cdkn1a* (*p21*) locus, leading to a robust induction of the *Cdkn1a* gene (Figure S1A-C in Additional file 1). This was accompanied by increased H3K4me3 levels at the *Cdkn1a* locus (Figure S1D in Additional file 1) as previously described [29]. To identify genes whose expression is regulated in response to doxorubicin treatment, we performed RNA-Seq experiments using cDNA libraries from ribosomal RNA-depleted total RNA in doxorubicin-treated and untreated WT ES cells. We identified 1,264 genes that significantly changed expression upon doxorubicin treatment (*P*-value <0.05, fold change ≥2.5) comprising 775 upregulated and 489 downregulated genes (Figure 1A; Additional file 2). The list included many genes previously implicated in the p53-dependent transcriptional response to doxorubicin [33,34] in both the upregulated *(Wnt9*, *Wnt3*, *Btg2*, *Cdkn1a*, *Mdm2*, *Bbc3*) and downregulated (*Sox2*, *Esrrb*, *Sall4*, *Zic3*, *n-Myc*, *Nanog*) gene sets (Figure 1A,B; Figure S2A in Additional file 3). These data confirm the validity of our data analysis pipeline.

H3K4me3 associates with active or poised promoters at a level dependent on gene activity [7,8]. Furthermore, transcriptional induction can lead to increased H3K4me3 at the activated gene promoter [35]. We asked how expression changes induced by doxorubicin affect H3K4me3 patterns at cognate promoters by performing H3K4me3 chromatin immunoprecipitation (ChIP) coupled to massively parallel DNA sequencing (ChIP-Seq) in WT ES cells that were untreated or treated with doxorubicin. Most promoters of upregulated genes are CpG-rich and displayed H3K4me3 enrichment before induction (Figure 1C), which is compatible with the notion that they are poised for rapid activation in response to external stimuli [36]. As expected, transcriptional induction by doxorubicin led to increased H3K4me3 levels at those promoters (Figure 1C). On the other hand, genes downregulated by doxorubicin treatment were characterized by decreased H3K4me3 (Figure S2A,B in Additional file 3), emphasizing the correlation between H3K4me3 and transcriptional activity.

In ES cells, Cfp1 plays a key role in establishing H3K4me3 at constitutively active loci, while its general contribution to H3K4me3 deposition at poised genes is minimal [28]. To explore how Cfp1 contributes to H3K4me3 dynamics in regulated gene expression, we analyzed H3K4me3 status at the *Cdkn1a* locus in response to doxorubicin in *Cfp1*^*−/−*^ ES cells by ChIP-quantitative PCR (ChIP-qPCR; Figure 1D). While we observed a robust increase in H3K4me3 at transcription start site (TSS)1, TSS2 and intron 1 upon doxorubicin treatment in WT ES cells, it was apparent that Cfp1-deficiency led to a severe defect in H3K4me3 accumulation at the *Cdkn1a* locus (Figure 1D). ChIP-qPCR using an antibody recognizing total histone H3 and a control IgG confirmed the specificity of this result (Figure S1E,F in Additional file 1). As expected, Cfp1-deficiency also led to decreased H3K4me3 near the TSS of constitutively active *Actb* and *Gapdh* gene loci (Figure 1D). We extended the analysis by performing H3K4me3 ChIP-Seq analyses in *Cfp1*^*−/−*^ ES cells treated or not with doxorubicin. Indeed, H3K4me3 did not increase at promoters of genes normally upregulated by doxorubicin treatment, but instead remained at basal levels in both untreated or doxorubicin-treated *Cfp1*^*−/−*^ ES cells (Figure 1E-H). This establishes that Cfp1 is instrumental in H3K4me3 deposition in response to an external stimulus in ES cells. On the other hand, genes downregulated by doxorubicin showed reduced H3K4me3 in untreated *Cfp1*^*−/−*^ compared to untreated WT ES cells and these levels did not drastically change upon doxorubicin treatment (Figure S2B-F in Additional file 3). Down-regulated genes are highly expressed in untreated ES cells (Figure S2G in Additional file 3) and therefore require Cfp1 for accurate H3K4me3 deposition at their promoters in normally proliferating mouse ES cells [28]. We conclude that upon transcriptional activation, H3K4me3 levels are enhanced at induced promoters in a Cfp1-dependent manner. Thus, Cfp1 not only plays a key role in determining proper H3K4me3 patterns in normally proliferating ES cells [28], but is also instrumental in targeting this modification to gene promoters upon transcriptional induction in response to external stimuli. These findings favor a model whereby the Cfp1/Set1-containing complex acts mainly downstream of transcriptional activation.

### Low H3K4me3 at promoters only mildly affects transcriptional induction output

**Figure 2:**
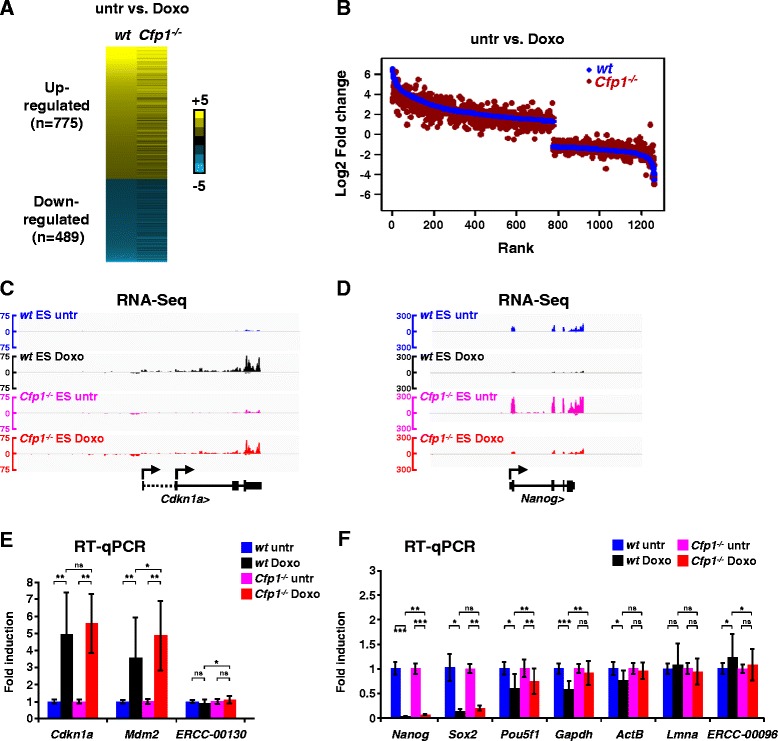
**Decreased H3K4me3 at regulated promoters mildly affects transcriptional output. (A)** Heatmap comparing gene expression changes (as log2 fold change) induced by doxorubicin treatment in WT and *Cfp1* ^*−/−*^ ES cells as determined by RNA-Seq. **(B)** Dot plot comparing gene expression differences in WT and *Cfp1* ^*−/−*^ ES cells. Genes were sorted on the basis of their log2 fold change upon doxorubicin treatment in WT ES cells (most upregulated gene on the far left, most downregulated gene on the far right). The log2 fold changes (untreated (untr) versus doxorubicin treatment (Doxo)) are plotted for WT (blue) and *Cfp1* ^*−/−*^ ES cells (red). **(C)** Genome browser screenshots representing RNA-Seq signal (as normalized read count) in WT ES cells (untreated in blue, treated in black) and *Cfp1* ^*−/−*^ (untreated in pink, treated in red) ES cells at the *Cdkn1a* locus. **(D)** Same as **(C)** for the *Nanog* locus. **(E)** Absolute quantification of *Cdkn1a* and *Mdm2* mRNA levels in WT (untreated in blue, doxorubicin-treated in black) and *Cfp1* ^*−/−*^ ES cells (untreated in pink, doxorubicin-treated in red) by RT-qPCR. Expression levels are normalized using an RNA spike-in standard (ERCC-00074) and then expressed as fold induction compared with the untreated value. The ERCC-00130 standard is shown as control. Data are represented as mean ± standard deviation, n = 4. *P*-values were calculated using two-tailed Student’s *t*-tests: *P < 0.05, **P < 0.01; *P* > 0.05 is not significant (ns). **(F)** Same as (E) for *Nanog*, *Sox2*, *Pou5f1*, *Gapdh*, and *Actb* mRNA levels. *Lmna* and ERCC-00096 are shown as controls.

Previous work established that lack of Cfp1-dependent H3K4me3 at constitutively active gene promoters does not drastically alter steady-state transcription in ES cells [28]. However, H3K4me3-mediated recruitment of Taf3 is reported to be crucial for transcriptional induction upon doxorubicin treatment in HCT116 cells [37]. We therefore tested whether altered H3K4me3 patterns affect the transcriptional response to doxorubicin. First, we checked that early response to doxorubicin was intact in *Cfp1*^*−/−*^ ES cells by confirming that p53 stabilization, phosphorylation and binding to the *Cdnkn1a* locus was indistinguishable between WT and *Cfp1*^*−/−*^ ES cells (Figure S3A,B in Additional file 4). We then performed RNA-Seq experiments in *Cfp1*^*−/−*^ ES cells treated or untreated with doxorubicin and looked for differential expression of genes that are inducible in WT ES cells. Many genes, including *Mdm2*, *Btg2* and *Wnt9a*, were induced appropriately in the mutant cells (Figure S3C in Additional file 4). Others appeared to be affected in their transcriptional response, but this effect was generally mild and gene-dependent, with very few genes showing complete lack of activation or repression (Figure 2A-C). We also observed minor defects in genes repressed by doxorubicin (Figure 2A,B), but confirmed that *Nanog* responded properly in both treated WT and treated *Cfp1*^*−/−*^ ES cells (Figure 2D). To exclude the possibility that Cfp1-deficiency affects global transcription in response to doxorubicin treatment, we performed absolute quantification RT-qPCR experiments. For this, we normalized transcript abundance to a spiked-in standard instead of an endogenous transcript (see Materials and methods for details) as described in [38]. In this setting, we also observe efficient *Cdkn1a* and *Mdm2* induction, as well as *Nanog* and *Sox2* repression in doxorubicin-treated WT and *Cfp1*^*−/−*^ ES cells (Figure 2E,F). Our results suggest that Cfp1-dependent H3K4me3 plays a minor role in ensuring appropriate expression of inducible genes. The biological significance of modest deregulation at specific genes is, however, uncertain.

### Cfp1 is crucial for H3K9 acetylation at regulated promoters

**Figure 3:**
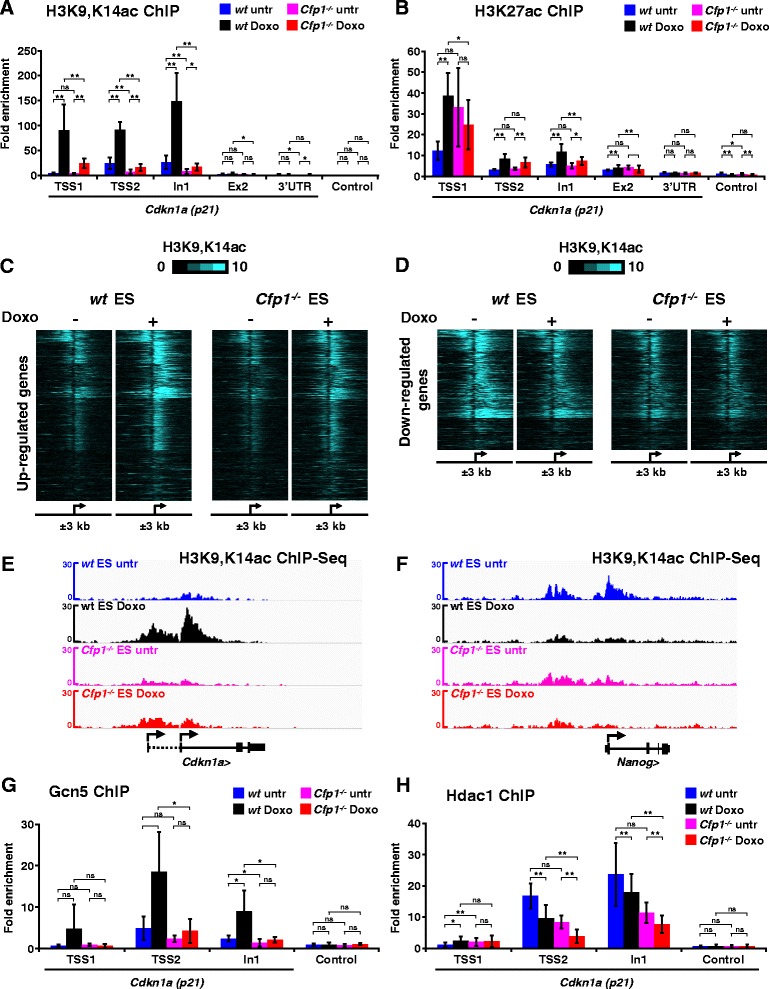
**Cfp1 is crucial for H3K9 acetylation at regulated promoters. (A)** ChIP-qPCR analysis of H3K9,K14ac enrichment at the *Cdkn1a* locus for untreated (untr) or doxorubicin-treated (Doxo) WT and *Cfp1* ^*−/−*^ ES cells. Results are expressed as fold enrichment relative to input DNA and a control intergenic region on chromosome 15. Control corresponds to an intergenic region on chromosome 5. Data are represented as mean ± standard deviation (SD), n = 3. *P*-values were calculated using two-tailed Student’s *t*-tests: *P < 0.05, **P < 0.01; *P* > 0.05 is not significant (ns). **(B)** Same as** (A)** for H3K27ac enrichment. Data are represented as mean ± SD, untr n = 2, Doxo n = 4. **(C)** Heatmap representation showing H3K9,K14ac normalized read count 3 kb upstream and downstream of TSSs of genes upregulated by doxorubicin treatment in WT ES cells (n = 755). **(D)** Same as **(C)** for downregulated genes (n = 489). **(E)** Genome browser screenshots representing H3K9,K14ac ChIP-Seq signal (as normalized read count) in WT (untreated in blue, treated in black) and *Cfp1* ^*−/−*^ (untreated in pink, treated in red) ES cells at the *Cdkn1a* locus. **(F)** Same as **(E)** for the *Nanog* locus. **(G)** Same as **(A)** for Gcn5 enrichment at the *Cdkn1a* locus. Data are represented as mean ± SD, n = 3. *P*-values were calculated using two-tailed Student’s *t*-tests: *P < 0.05, **P < 0.01, *P* > 0.05 (ns). **(H)** Same as **(G)** for Hdac1 enrichment at the *Cdkn1a* locus. Data are represented as mean ± SD, n = 3. *P*-values were calculated using two-tailed Student’s *t*-tests: *P < 0.05, **P < 0.01, *P* > 0.05 (ns).

As lack of Cfp1 led to impaired H3K4me3 accumulation at doxorubicin-regulated promoters, we investigated whether other aspects of the chromatin landscape were also affected. Several reports have linked H3K4me3 levels at TSSs with histone H3 acetylation [29,35,39,40]. Using an antibody directed against H3K9,K14ac, we observed increased histone acetylation at the *Cdkn1a* locus upon doxorubicin treatment in WT ES cells, but this effect was absent in *Cfp1*^*−/−*^ cells (Figure 3A). A possible explanation is that histone H3 acetylation requires Cfp1-dependent H3K4me3 deposition at the induced TSSs. This effect appears specific to H3K9,K14 as H3K27 acetylation levels were unaffected by absence of Cfp1 (Figure 3B). Furthermore, antibodies specifically directed against H3K9 or H3K14 acetylation revealed that Cfp1-deficiency more severely impacts H3K9 rather than H3K14 acetylation (Figure S4A,B in Additional file 5). To test whether Cfp1-dependent H3K4me3 at doxorubicin-regulated promoters is generally associated with H3K9,K14 acetylation, we performed ChIP-Seq experiments. In WT ES cells we observed increased TSS histone acetylation at genes induced by doxorubicin, but acetylation was severely reduced in *Cfp1*^*−/−*^ ES cells (Figure 3C,E; Figure S4C in Additional file 5). At down-regulated promoters, H3K9,K14 acetylation was decreased in treated WT ES cells, but its levels appear low in both untreated and doxorubicin-treated *Cfp1*^*−/−*^ cells (Figure 3D,F; Figure S4D in Additional file 5). There is therefore a strong similarity between H3K4me3 and H3K9,K14ac patterns at doxorubicin-regulated promoters, as levels of both are directly related to transcriptional activity. Interestingly, lack of Cfp1 impacts both modifications at this subset of genes, strongly suggesting co-dependency.

H3K4me3 can act as a binding platform that attracts HAT complexes. In particular, the Gcn5-containing SAGA complex can be targeted to H3K4me3 nucleosomes by the adaptor subunit Sgf29 [41]. Consistent with this model, we observed that decreased H3K4me3 at the *Cdkn1a* promoter is associated with decreased Gcn5 binding at the same genomic location (Figure 3G), which is likely to account for decreased H3K9 acetylation. Furthermore, we observed that reduced histone acetylation at the *Cdkn1a* locus in *Cfp1*^*−/−*^ ES cells was not due to increased binding of histone deacetylase 1 (Hdac1; Figure 3H). We conclude that H3K4me3 defects caused by Cfp1-deficiency at promoters affect overall H3K9 acetylation dynamics, probably by directly impairing H3K4me3-mediated HAT recruitment.

### H3K9ac levels follow H3K4me3 at Cfp1-regulated regions independent of gene induction

**Figure 4:**
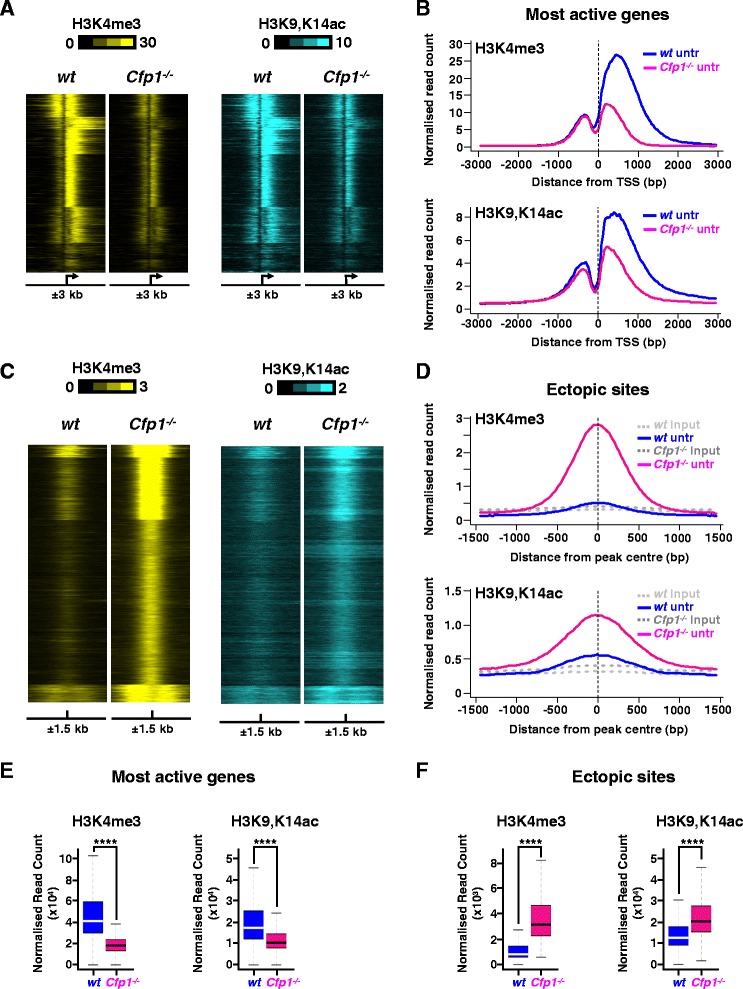
**H3K9 acetylation is altered at Cfp1-regulated regions. (A)** Heatmap showing H3K4me3 (yellow) or H3K9,K14ac (blue) normalized read count at TSSs for the most active genes in WT ES cells (n = 2,500). Signal is displayed from −3 kb to +3 kb surrounding each annotated TSS for untreated WT or *Cfp1* ^*−/−*^ ES cells. **(B)** Average profile showing H3K4me3 or H3K9,K14ac normalized read count at TSSs for the most active genes in WT ES cells (n = 2,500). Signal is displayed from −3 kb to +3 kb surrounding each annotated TSS for untreated WT or *Cfp1* ^*−/−*^ ES cells. **(C)** Same as **(A)** for regions aberrantly accumulating H3K4me3 in absence of Cfp1 (n = 14,208). Signal is displayed from −1.5 kb to +1.5 kb surrounding the center of the peak for each region for untreated WT or *Cfp1* ^*−/−*^ ES cells. **(D)** Average profile showing H3K4me3 or H3K9,K14ac normalized read count for regions aberrantly accumulating H3K4me3 in absence of Cfp1 (n = 14,208). Signal is displayed from −1.5 kb to +1.5 kb surrounding the centre of the peak. Input DNA is plotted as a reference. **(E)** Comparison of H3K4me3 or H3K9,K14ac normalized read density at TSSs of the most active genes (n = 2,500), from 3 kb upstream to 3 kb downstream. Box plots show the central 50% of the data (filled box), the median (central bisecting line) and 1.5× the interquartile range (whiskers). *P*-values were calculated using two-tailed unpaired Wilcoxon tests: *P < 0.05, **P < 0.01, ***P < 0.001, ****P < 0.0001. **(F)** Same as **(E)** for H3K4me3 or H3K9,K14ac normalized read density at regions aberrantly accumulating H3K4me3 in absence of Cfp1 (n = 14,208), from 1.5 kb upstream to 1.5 kb downstream.

Cfp1 influences H3K4me3 deposition at many genomic locations spread throughout the mouse ES cell genome and particularly at active gene promoters [28]. We tested whether Cfp1-deficiency affects histone acetylation at non-doxorubicin-inducible promoters by examining H3K4me3 patterns at the most active genes in WT ES cells as identified in our RNA-Seq dataset (n = 2,500; Additional file 6). The results confirm that lack of Cfp1 leads to severely reduced H3K4me3 levels, mainly located downstream of the TSS (Figure 4A,B,E) and that this is accompanied by a reduction in H3K9,K14ac as determined by ChIP-Seq (Figure 4A,B,E). Similar results were obtained for this gene set when comparing doxorubicin-treated WT and *Cfp1*^*−/−*^ ES cells (Figure S5A,B,E in Additional file 7). The findings further support a mechanistic link between Cfp1-dependent H3K4me3 and H3K9ac at TSSs in normally cycling mouse ES cells.

Absence of Cfp1 in mouse ES cells has been shown to cause aberrant accumulation of H3K4me3 at many non-promoter regions [28]. We tested the possibility that these ectopic sites of H3K4me3 accumulation are also associated with increased H3K9ac. For this, we compared H3K4me3 and H3K9,K14ac density in WT and *Cfp1*^*−/−*^ ES cells at those sites where H3K4me3 accumulates in the absence of Cfp1 (Additional file 8). The results confirmed that, on average, increased H3K4me3 at those regions is matched by increased H3K9,K14ac levels (Figure 4C,D,F). Furthermore, it was obvious that regions that accumulated the most H3K4me3 in *Cfp1*^*−/−*^ ES cells also showed highest H3K9,K14ac enrichment (Figure 4C,D,F). Again, this was confirmed when comparing doxorubicin-treated WT and *Cfp1*^*−/−*^ ES cells (Figure S5C,D,F in Additional file 7). Some of the regions that accumulate H3K4me3 in cells lacking Cfp1 correspond to sites occupied by Ctcf or Cohesin [28]. We therefore analyzed both H3K4me3 and H3K9,K14ac distributions at selected binding sites for those two factors in WT and *Cfp1*^*−/−*^ ES cells [42] (Additional files 9 and 10). Lack of Cfp1 leads to increased H3K4me3 and this is associated with an increase in H3K9,K14ac (Additional files 11 and 12). We conclude that the altered H3K4me3 pattern observed in mouse ES cells in the absence of Cfp1 is accompanied by an alteration in H3K9ac deposition. This suggests a mechanistic co-dependency for these two histone marks, both of which are consistently associated with transcriptional activity.

### Decreased H3K4me3 and H3K9ac impact transcriptional accuracy upon induction

**Figure 5:**
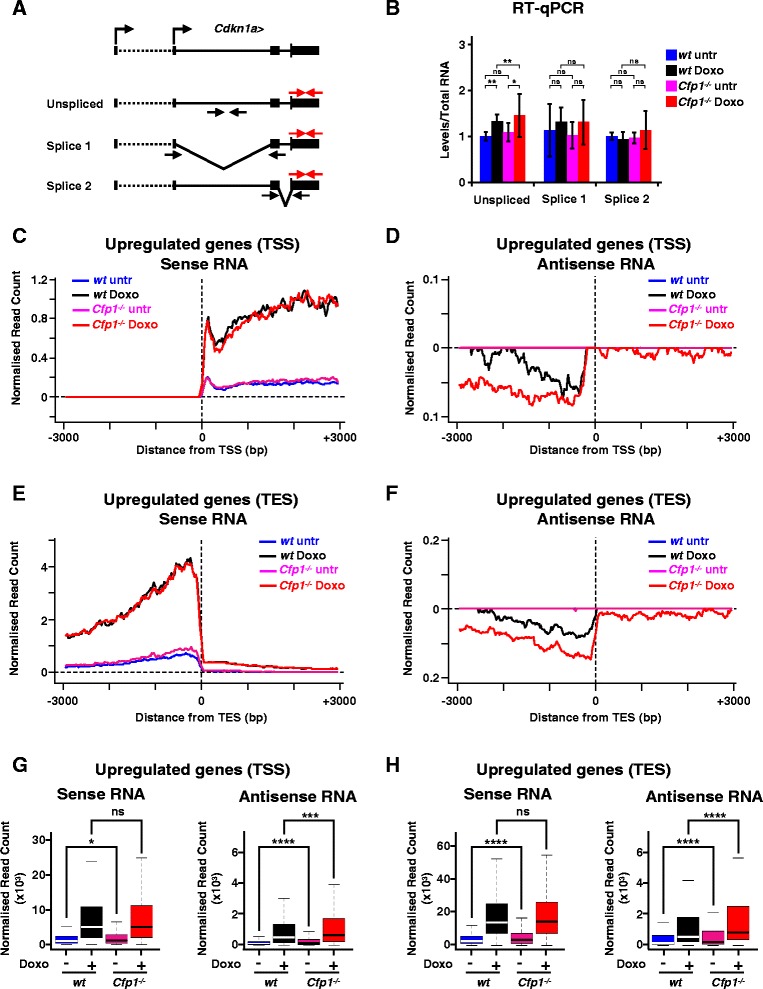
**Decreased H3K4me3 and H3K9ac influence transcriptional accuracy upon induction. (A)** Schematic representation of the qPCR strategy used to quantify various *Cdkn1a* mRNA splicing products. Total RNA is detected using primers on 3’ UTRs (shown in red). **(B)** RT-qPCR analysis of *Cdkn1a* splicing products. *P*-values were calculated using two-tailed Student’s *t*-tests: *P < 0.05, **P < 0.01, *P* > 0.05 is not significant (ns). **(C)** Average profile showing sense RNA-Seq normalized read count at TSSs of genes induced by doxorubicin treatment in WT ES cells (n = 755). Signal is displayed from −3 kb to +3 kb surrounding each annotated TSS. **(D)** Same as **(C)** for antisense RNA-Seq normalized read count (note that sense and antisense transcripts are plotted on a different scale). **(E)** Average profile showing sense RNA-Seq normalized read count at transcription end sites (TESs) of genes induced by doxorubicin treatment in WT ES cells (n = 755). Signal is displayed from −3 kb to +3 kb surrounding each annotated TES. **(F)** Same as **(E)** for antisense RNA-Seq normalized read count (note that sense and antisense transcripts are plotted on a different scale). **(G)** Comparison of sense or antisense RNA-Seq normalized read density at TSSs of upregulated genes (n = 755), from 3 kb upstream to 3 kb downstream, in WT or *Cfp1* ^*−/−*^ ES cells, treated or not with doxorubicin as indicated. Box plots show the central 50% of the data (filled box), the median (central bisecting line) and 1.5× the interquartile range (whiskers). *P*-values were calculated using two-tailed unpaired Wilcoxon tests: *P < 0.05, **P < 0.01, ***P < 0.001, ****P < 0.0001. **(H)** Same as **(G)** for TESs of upregulated genes (n = 755).

Given the subtle effect of altered promoter H3K4me3 and H3K9ac on the transcriptional output of inducible genes, we tested whether other aspects of gene expression are affected by the altered chromatin landscape. First we examined RNA processing, as H3K4me3 is proposed to facilitate splicing factor loading [43]. We compared splicing efficiency for the *Cdkn1a* transcript upon induction in WT and *Cfp1*^*−/−*^ ES cells. We detected neither accumulation of the unspliced pre-mRNA nor reduction in a particular splicing product when comparing doxorubicin-treated WT and *Cfp1*^*−/−*^ ES cells (Figure 5A,B). This agrees with a previous observation that decreased H3K4me3 at constitutively active genes does not drastically alter splicing in normally cycling ES cells [28].

Antisense transcription is also a feature of gene expression thought to be affected by H3K4 methylation [44-46]. Active mammalian transcription units produce small sense and antisense RNA molecules at their TSSs and transcription end sites (TESs) [47,48]. We investigated levels of these transcripts at genes regulated by doxorubicin in WT and *Cfp1*^*−/−*^ ES cells using strand-specific RNA-Seq datasets. As expected, upon induction sense RNA accumulated both downstream of the TSS and upstream of the TES (Figure 5C,D,G,H) and profiles were strongly similar when comparing doxorubicin-treated WT and *Cfp1*^*−/−*^ ES cells. Induced genes also displayed increased divergent antisense transcription at both TSSs and TESs, albeit at much lower abundance, but this was strikingly elevated in *Cfp1*^*−/−*^ ES cells (Figure 5C,D,G,H). The results suggest that Cpf1-dependent increases in H3K4me3 and H3K9ac can play a role in regulating TSS- and TES-associated small RNA transcription upon gene induction. The effect is marginal or non-significant at genes downregulated by doxorubicin (Additional file 13). We propose that Cfp1-dependent H3K4me3 and H3K9ac depositions contribute to accurate RNA production at the start and end of genes upon transcriptional induction.

## Discussion

### Understanding H3K4me3 deposition at gene promoters

Cfp1 is required for accurate H3K4me3 deposition at constitutively active gene promoters in mouse ES cells [28]. Here we show that Cfp1 also plays an important role in targeting this modification upon transcriptional induction. This favors a model in which the Cfp1/Set1 complex acts mainly downstream of the transcriptional apparatus, similar to yeast [5,6,49]. The data suggest a scenario whereby high H3K4me3 levels at promoters of genes regulated by external stimuli result from a multistep process. In the unstimulated state H3K4me3 is low and the promoter is poised for activation. Cfp1 seems to play little role in this 'priming' process. When transcription is induced, however, Cfp1 is instrumental in causing the striking increase in H3K4me3 levels at the TSS. Similarly, Cfp1 is dispensable for H3K4me3 at bivalent genes [28], which are kept silenced in ES cells but are thought to be poised for activation by developmental cues [13,14,50]. The histone methyl transferase Mll2 is largely responsible for H3K4me3 deposition at bivalent promoters [23]. Thus, different H3K4 methyl-transferases play distinct roles in shaping the chromatin landscape, suggesting that histone modification patterns are the results of an intricate network of enzymatic activities. Variation in these histone modifications may create alternative binding surfaces for potential histone tail readers, leading to context-dependent outcomes. According to this scenario, histone modifications do not act as simple on/off switches, but instead create molecular signaling gradients, allowing for subtle regulation of particular genomic loci in various conditions. More quantitative measurements of chromatin features are needed to fully appreciate their complexity and relationship to cell commitment and disease.

### H3K4me3 and H3K9ac deposition are linked in mouse embryonic stem cells

A striking finding from our study is that alterations in Cfp1-dependent H3K4me3 deposition are accompanied by parallel defects in H3K9ac deposition. This is true for regions that either lose or gain H3K4me3 in the absence of Cfp1. An attractive possibility is that H3K9 acetylation is downstream of H3K4me3, and, together with previous reports, points to a molecular function for H3K4me3 in targeting H3K9 acetylation. H3K4me3 and H3K9ac coincide at many gene promoters [8,51-53], but we observe that these two marks are mechanistically co-dependent. H3K4me3 was suggested to facilitate H3K9 acetylation [40] and Sgf29, a subunit of the SAGA complex, can bind H3K4me3 and facilitate recruitment of the Gcn5 HAT [41]. Accordingly, we find that decreased H3K4me3 in *Cfp1*^*−/−*^ ES cells leads to reduced Gcn5 recruitment at the *Cdkn1a* promoter. Furthermore, aberrant H3K4me3 accumulation at ectopic sites in the absence of Cfp1 coincides with enhanced H3K9ac deposition at the same loci. These findings do not favor a hypothesis where Cfp1 functions in an as yet uncharacterized HAT complex, but this possibility cannot be formally excluded. We favor a simpler model whereby Cfp1-dependent enhancement of H3K4me3 levels at TSSs contributes to recruitment of HATs, which increase H3K9 acetylation. Interestingly, the inverse relationship, whereby acetylation of histone H3 targets the Set1 complex to chromatin, has been observed biochemically, although the acetylation affects residues other than K9 [54]. Further studies of the functional interplay between histone marks will undoubtedly shed valuable light on the biological significance of histone modifications.

### H3K4me3 and H3K9ac function in a context-dependent manner

Reduced H3K4me3 at a TSS does not affect steady-state transcription in ES cells [28,29]. We now extend this observation and show that H3K4me3 is largely dispensable for regulated gene expression in this cell type. *In vitro*, H3K4me3 stimulates assembly of the pre-initiation complex as well as p53- and p300-dependent transcription [37,54]. Furthermore, Taf3, a TBP-associated protein that can bind H3K4me3, selectively regulates a p53 target gene induced by doxorubicin in colon cancer cells [37]. On the other hand, depletion of Mll2 in ES cells, which is responsible for H3K4me3 deposition at bivalent genes, has little effect on their rapid transcriptional induction [23]. Cfp1/Set1 associates with most CGI promoters in mouse brain and is required for regulated gene expression in colon cancer cells [12,54]. In pluripotent cells, however, Cfp1 is only necessary for H3K4me3 deposition at active genes and does not strongly affect either steady-state or regulated gene expression ([28] and this study). We conclude that the impact of H3K4me3 deposition on nucleosomes is highly context-dependent and that this modification may play gene- and/or cell type-specific roles.

Our data also imply that high level H3K9 acetylation is not required for either steady-state or regulated gene expression in ES cells. The association between histone acetylation and active transcription is well established [55,56], but it has been reported that histone deacetylases, which remove acetylation, are also enriched at active gene promoters [40]. It appears that two of the marks most prominently associated with transcriptional initiation are not necessary for either steady-state or regulated gene expression in mouse ES cells. It is possible that their functional significance is gene-specific and/or context-dependent. Alternatively, there may be general functions for these marks that have so far escaped detection. In yeast, Set1 deletion has effects on regulating antisense transcription [44-46]. We observed aberrant antisense transcription at TSSs and TESs of genes induced by doxorubicin, suggesting that H3K4me3 and/or H3K9ac can regulate complex aspects of mRNA biogenesis. These transcripts are low in abundance, however, and do not seem to critically influence mRNA levels. A second possibility is that H3K4me3 and/or H3K9ac are required to help resolve potentially toxic R-loops that arise at transcriptionally active CpG rich promoters in mammals [57]. Thirdly, the presence of these marks may fine-tune RNA polymerase II dynamics in ways that are not easily caught by genome-wide studies, which provide only an averaged snapshot of the cell population.

Our data could also fit with the idea that histone modifications mainly modulate nucleosome dynamics [58,59]. ES cells reportedly have an intrinsically accessible, dynamic chromatin structure, which is believed to contribute to their pluripotency [4,60]. Such an atypical structure could be robust enough to sustain key processes such as transcription, for which histone modification only acts redundantly. Upon lineage commitment, however, a more restricted chromatin state may be acquired [4] for which histone modification-mediated processes become necessary. One also needs to stay open to the idea that histone marks strongly associated with promoters or coding regions can play roles outside transcription. For example, H3K4me3, together with insulator proteins, demarcates the border of physical domains and might thus be important for shaping the three-dimensional organization of the nucleus [61] and H3K36me3 has recently been implicated in regulation of DNA repair [62]. There is still much to understand regarding the role of chromatin in complex genome organization and function.

## Conclusions

We show that Cfp1 is required for H3K4me3 accumulation at promoters upon rapid transcriptional induction, linking chromatin structure and transcriptional activity. We also uncover a strong relationship between Cfp1-dependant H3K4me3 accumulation and accurate H3K9ac deposition. As defects in H3K4me3 and H3K9ac accumulation at promoters do not drastically alter steady-state or regulated expression of the associated gene, our results suggest that those two histone modifications function downstream of the transcriptional apparatus and could potentially regulate small RNA levels around transcription unit boundaries.

## Materials and methods

### Embryonic stem cell culture

ES cells were grown in gelatinized dishes in Glasgow MEM (Gibco Life Technologies Ltd, Paisley, UK) supplemented with 10% fetal bovine serum (Hyclone GE Healthcare Bio-Sciences AB, Uppsala, Sweden), 1× MEM nonessential amino acids, 1 mM sodium pyruvate, 50 μM 2-mercaptoethanol (Gibco) and LIF (Leukemia Inhibitory Factor). E14TG2a ES cells were used as WT ES and *Cfp1*^*−/−*^ ES cells have been described [32]. Doxorubicin was purchased from Sigma (Gillingham, UK) and used at a final concentration of 1 μM for 6 hours.

### Chromatin immunoprecipitation

ES cells were crosslinked with 1% formaldehyde at room temperature for 10 minutes, followed by 5-minute incubation with 125 mM glycine at room temperature. Cells were washed in phosphate-buffered saline, flash frozen in liquid nitrogen and stored at −80°C prior to use. Cell pellets were thawed on ice, resuspended in lysis buffer 1 (50 mM Hepes KOH, pH 7.9, 140 mM NaCl, 1 mM EDTA, 10% glycerol, 0.5% NP-40, 0.25% Triton X-100) and incubated for 10 minutes on a rotator at 4°C. Cells were washed in TE (10 mM Tris–HCl, pH 8, 1 mM EDTA).

For histone modifications and RNA polymerase II ChIP, cells were resuspended in TE containing 0.5% SDS, incubated on ice for 10 minutes and sonicated using a Bioruptor (Diagenode, Seraing, Belgium) on high setting for 18 cycles of 30 s (30 s pause between pulses). The sonicated extract was centrifuged at 20,000 g for 10 minutes at 4°C and the supernatant diluted 5-fold with ChIP dilution buffer (20 mM Tris–HCl, pH 8, 150 mM NaCl, 1% Triton X-100, 1 mM EDTA).

For Gcn5, CBP, Hdac1 and p53S15P ChIP, cells were sonicated in 20 mM Tris–HCl, pH 8, 150 mM NaCl, 1% Triton X-100, 1 mM EDTA, 0.1% SDS for 60 cycles of 30 s (30 s pause between pulses).

Sonicated chromatin was incubated overnight with the appropriate antibody and immune complexes were precipitated using protein A-Sepharose (GE Healthcare Bio-Sciences AB, Uppsala, Sweden) pre-blocked with bovine serum albumin and yeast tRNA, for 30 minutes at 4°C. Complexes were washed three times with 20 mM Tris–HCl, pH 8, 150 mM NaCl, 1% Triton X-100, 1 mM EDTA, 0.1% SDS, once with 20 mM Tris–HCl, pH 8, 500 mM NaCl, 1% Triton X-100, 1 mM EDTA, 0.1% SDS, and once with TE 1X. Bound complexes were eluted from the beads with TE containing 1% SDS by heating at 65°C for 1 h. Samples were diluted to 0.5% SDS with TE, then RNase A and NaCl (final concentration 0.3 M) were added and samples incubated for 4 h to overnight at 65°C to reverse crosslinking. Samples were treated with proteinase K (100 μg/ml) for 1 to 2 h at 55°C, phenol extracted and ethanol precipitated.

### Strand-specific RNA-Seq

Cells were lysed in TRI reagent (Sigma). Total RNA was then extracted with chloroform, precipitated with isopropanol and DNAse treated (DNA-free, Ambion Life Technologies Ltd, Paisley, UK). Ribosomal RNA from 5 μg of total RNA was depleted using Ribo-Zero kit (Epicentre, Madison, WI, USA). Strand specific RNA-Seq libraries were prepared using Script-Seq V2 RNA-Seq library preparation kit (Epicentre) according to the manufacturer’s instructions.

### Preparation of ChIP DNA for Illumina sequencing

ChIP DNA was end repaired by incubation at 20°C for 30 minutes with 3 U of T4 DNA Polymerase (NEB, Hitchin, UK), 10 U of Polynucleotide Kinase (NEB), 2 U DNA Polymerase I Large (Klenow) fragment (NEB), 1× T4 DNA ligase reaction buffer (NEB) and 400 nM dNTPs. The enzymes were then heat inactivated at 75°C for 20 minutes, after which the DNA was ethanol precipitated. A tail of 'A' bases was added to the 3’ ends of the DNA by incubation with 5 U Klenow Fragment (3’-5’ exo-; NEB), 200 nM dATP and 1X buffer 2 (NEB) at 37°C for 30 minutes. The enzymes were heat inactivated and cleaned up as before. Barcoded Ilumina paired end adaptors were then ligated to the processed ChIP DNA by incubation with 300 U of T4 DNA ligase (NEB), 1× T4 DNA ligase buffer (NEB), 7.5% PEG-6000 and 2 pmol of annealed Illumina adaptors for 3 h at room temperature. Ligated DNA was purified using MinElute PCR columns (Qiagen, Hilden, Germany) and eluted in 10 μl water.

### Library preparation and Illumina sequencing

Ligated DNA was amplified by 16 to 18 cycles of PCR with primers complementary to the adaptor sequences and Phusion 2x premix (Finnzymes, Waltham, MA, USA). The DNA was purified using QIAquick PCR Purification columns (Qiagen). The purified DNA was captured on an Illumina flow cell for cluster generation. Libraries were sequenced using an Illumina HiSeq to generate paired-end 75 bp reads. For ChIP-Seq, six samples were multiplexed. Paired-end sequence reads were demultiplexed and mapped to the mouse genome (NCBIm37) using BWA version 0.5.9-r16 [63]. Uniquely aligned reads were selected for further analysis and duplicate reads were removed for H3K9,K14ac ChIP-Seq samples (duplication was very low in H3K4me3 samples). For H3K4me3, sequencing from two biological replicates was combined in a single file. For RNA-Seq, eight samples were multiplexed. Paired-end sequence reads were mapped to the mouse genome (NCBIm37) using STAR version 2.20c_r200 [64] to allow spliced alignments. Biological replicates (two for each condition) were kept separate for differential expression analysis but were merged to generate average profiles.

### RNA expression analysis

A GTF file containing ensEMBL mouse transcript information (release 67) was used to generate a count table with HTSeq-count [65] with the following parameters: −-stranded yes -mode union -type exon. These options summarize unambiguous read counts, on the sense strand, over entire gene units using all exons. Genes differentially expressed upon doxorubicin treatment in WT ES cells were identified using the Bioconductor package DESeq [66]. Genes with adjusted *P*-value <0.05 and fold change ≥2.5 were identified as differentially expressed. Genes with a base mean count <200 (empirically determined) were removed from the list as they correspond to genes weakly expressed in both conditions. The corresponding differential expression heatmap (Figure 2A) was generated using Java Treeview [67]. A normalized count table was used to determine expression values (Figure S2G in Additional file 3) and identify the 2,500 most active genes in mouse ES cells (Figure 4) by correcting WT raw read counts for gene length. Average profile plots of sense and antisense transcription (Figure 5C-F) were generated in an identical manner to ChIP-Seq occupancy plots (see below) using normalized bigwig files for each strand (based on direction of paired end alignment).

### Average profile and heatmap analysis of ChIP-seq occupancy

All read depths were normalized to the total number of uniquely mapped reads in each experiment. ChIP-seq density was determined by calculating the average number of hits per base in 100 bp windows with a 20 bp slide using normalized bigwig files over a 6 kb interval centered on the TSS (for promoters) or a 3 kb interval over the center of each peak (for non-promoter regions) using custom Perl and R scripts interfaced with the Galaxy server [68,69], which are available upon request. Average profiles were generated by plotting the median value in each window for each sample. For heat maps, the sliding window file obtained from WT ES cells was subjected to k-means clustering using Cluster 3.0 [70]. Results from clustering were used to rank sliding windows files for all other samples accordingly. Clustered heatmaps were generated using Java Treeview.

### RNA extraction and RT-qPCR

Cells were lysed in TRI reagent (Sigma). RNA was then extracted with chloroform, precipitated with isopropanol and DNAse treated (DNA-free, Ambion). Total RNA (2 μg) was used per reverse transcription reaction. RNA was denatured in the presence of 2 μg of random hexamers (Invitrogen Life Technologies Ltd, Paisley, UK) for 5 minutes at 75°C, and reverse transcribed in a final volume of 40 μl with 200 U of SuperScript II (Invitrogen) at 42°C overnight followed by heat inactivation at 70°C for 15 minutes. Synthesized complementary DNAs were diluted in 300 μl of water and stored at −20°C until used.

Absolute quantification experiments were performed essentially as described in [39]. Briefly, 5.10^5^ cells were lysed in 1 ml TRI reagent (Sigma) and 1 μl of a 1:20 dilution of ERCC Spike-in Mix#1 (Life Technologies Ltd, Paisley, UK) was added to each sample. After RNA extraction and reverse transcription, cDNAs were analysed by RT-qPCR. Transcript abundance were normalized to ERCC-00074 spike-in standard. ERCC-00096 and ERCC-00130 were also used as secondary controls.

### Protein extraction and western blotting

Cells were lysed in RIPA buffer (25 mM Tris–HCl, pH 7.4, 150 mM NaCl, 1% NP-40, 1% sodium deoxycholate) plus protease inhibitors and sonicated with a Bioruptor (Diagenode) for 10 cycles (30 s each cycle). Whole cell lysate were subjected to SDS-PAGE and transferred on nitrocellulose.

### Antibodies

All antibodies are listed in Additional file 14.

### Oligonucleotides

Oligonucleotides used for ChIP-qPCR, RT-qPCR and splicing analysis are listed in Additional file 15.

### Data access

High throughput sequencing data (ChIP-Seq and RNA-Seq) have been deposited in the Gene Expression Omnibus (GEO) under the accession number GSE53492.

## Additional files

Additional file 1: Figure S1. Embryonic stem cell response to doxorubicin. Additional file 2: Table S1. Differentially expressed genes comparing WT versus WT doxorubicin-treated cells (*P* > 0.05, fold change ≥2.5). Additional file 3: Figure S2. Cfp1 regulates H3K4me3 at promoters of genes responding to doxorubicin in ES cells. Additional file 4: Figure S3. Impact of decreased H3K4me3 at regulated promoters on transcriptional output. Additional file 5: Figure S4. Cfp1-deficiency associates with decreased H3 acetylation at regulated promoters. Additional file 6: Table S2. The 2,500 most expressed gene in WT ES cells. Additional file 7: Figure S5. H3K9 acetylation is altered at Cfp1-regulated H3K4me3 binding sites in doxorubicin-treated cells. Additional file 8: Table S3. Coordinates for ectopic H3K4me3 peaks. Additional file 9: Table S4. Subset of Ctcf binding sites (from [42]). Additional file 10: Table S5. Subset of Smc1a binding sites (from [42]). Additional file 11: Figure S6. H3K9 acetylation is altered at Ctcf binding sites in the absence of Cfp1. Additional file 12: Figure S7. H3K9 acetylation is altered at cohesin binding sites in the absence of Cfp1. Additional file 13: Figure S8. Transcription at TSSs and TESs of genes downregulated by doxorubicin. Additional file 14: Table S6. List of antibodies used in the study. Additional file 15: Table S7. List of oligonucleotides used in the study.

## Abbreviations

bp: base pair
CGI: CpG island
ChIP: chromatin immunoprecipitation
ES: embryonic stem
HAT: histone acetyltransferase
qPCR: quantitative polymerase chain reaction
TES: transcription end site
TSS: transcription start site
UTR: untranslated region
WT: wild type
