# Supplementary figures and images for: Cfp1 is required for gene expression-dependent H3K4 trimethylation and H3K9 acetylation in embryonic stem cells
Source: Genome Biol. 2014 Sep 4;15(9):451. doi: 10.1186/s13059-014-0451-x (PMC4189735; doi:10.1186/s13059-014-0451-x)

Clouaire et al. **Additional File 1. Figure S1**

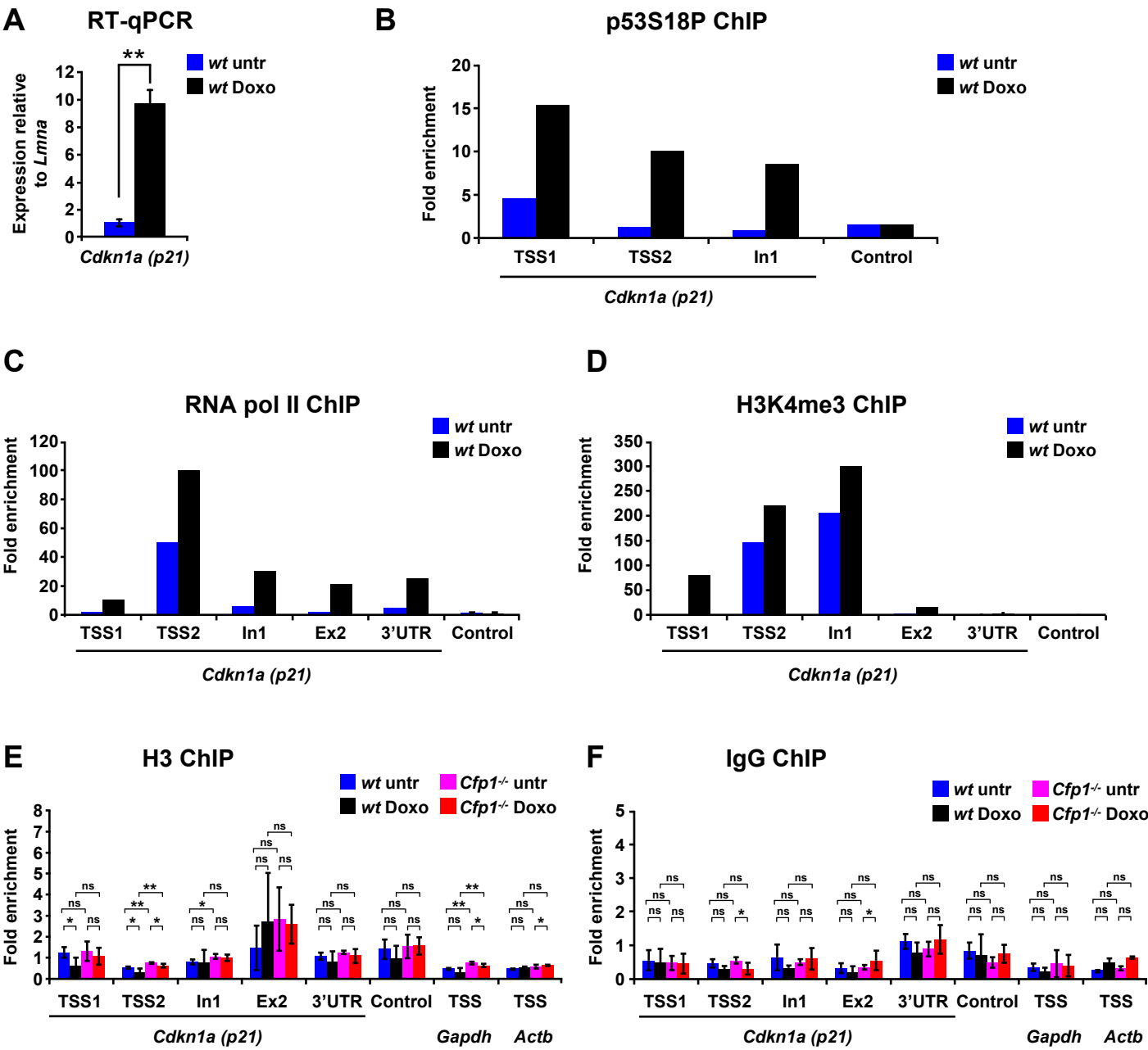

Supplement: Additional file 1: Figure S1. — Embryonic stem cell response to doxorubicin. [file 13059_2014_451_MOESM1_ESM.pdf]

Clouaire et al. **Additional File 3. Figure S2.**

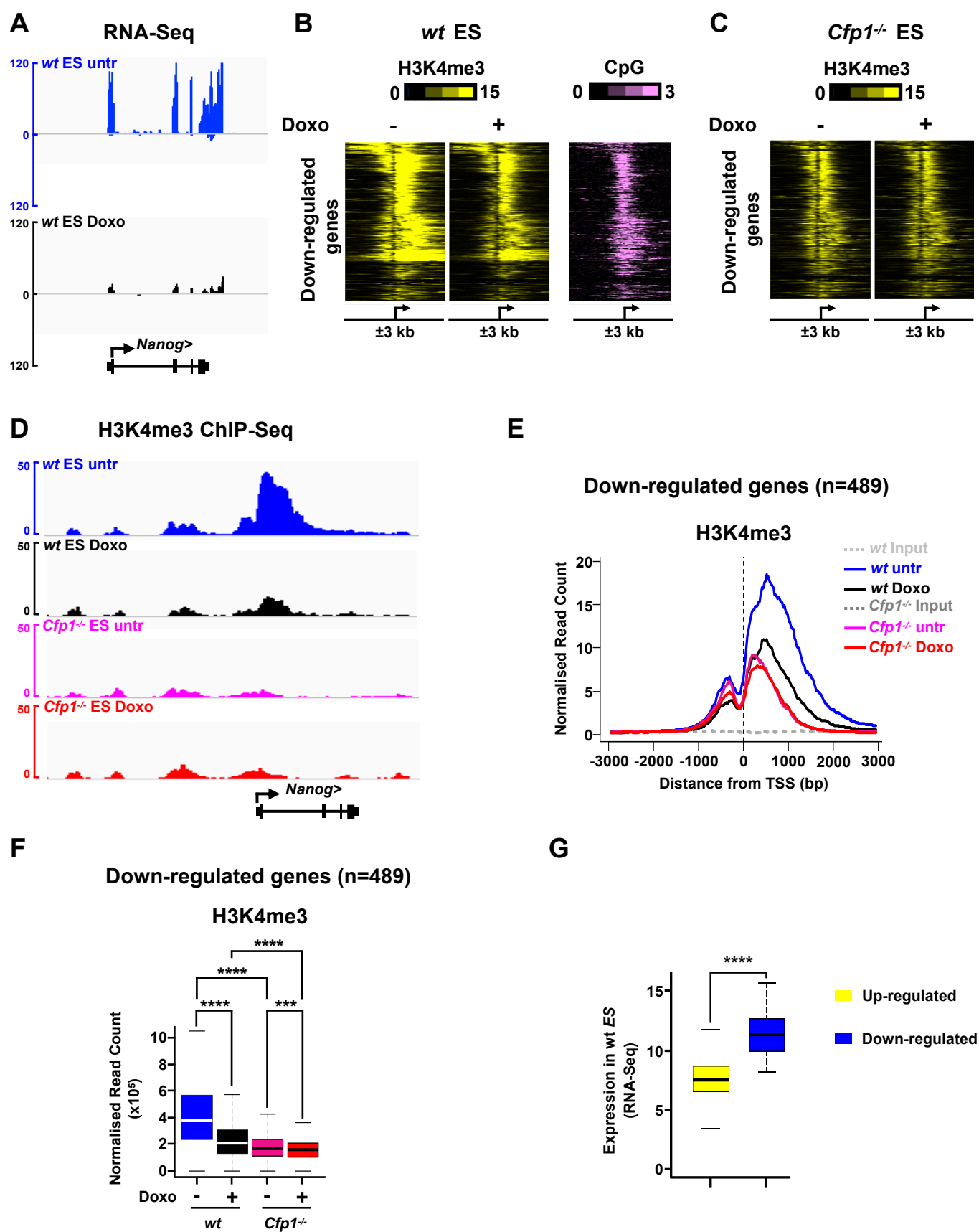

Supplement: Additional file 3: Figure S2. — Cfp1 regulates H3K4me3 at promoters of genes responding to doxorubicin in ES cells. [file 13059_2014_451_MOESM3_ESM.pdf]

Clouaire et al. **Additional File 4. Figure S3.**

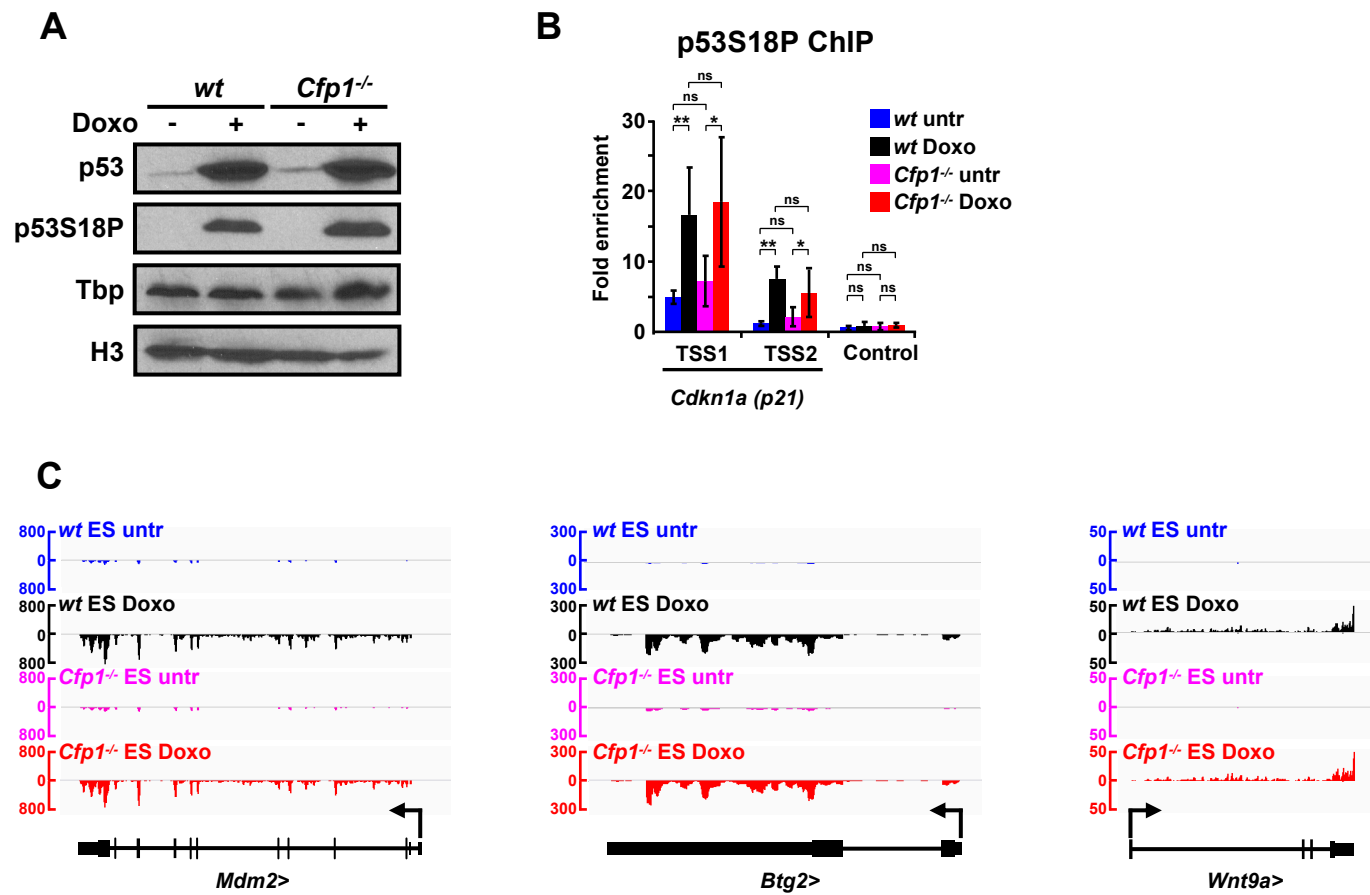

Supplement: Additional file 4: Figure S3. — Impact of decreased H3K4me3 at regulated promoters on transcriptional output. [file 13059_2014_451_MOESM4_ESM.pdf]

Clouaire et al. **Additional File 5. Figure S4**

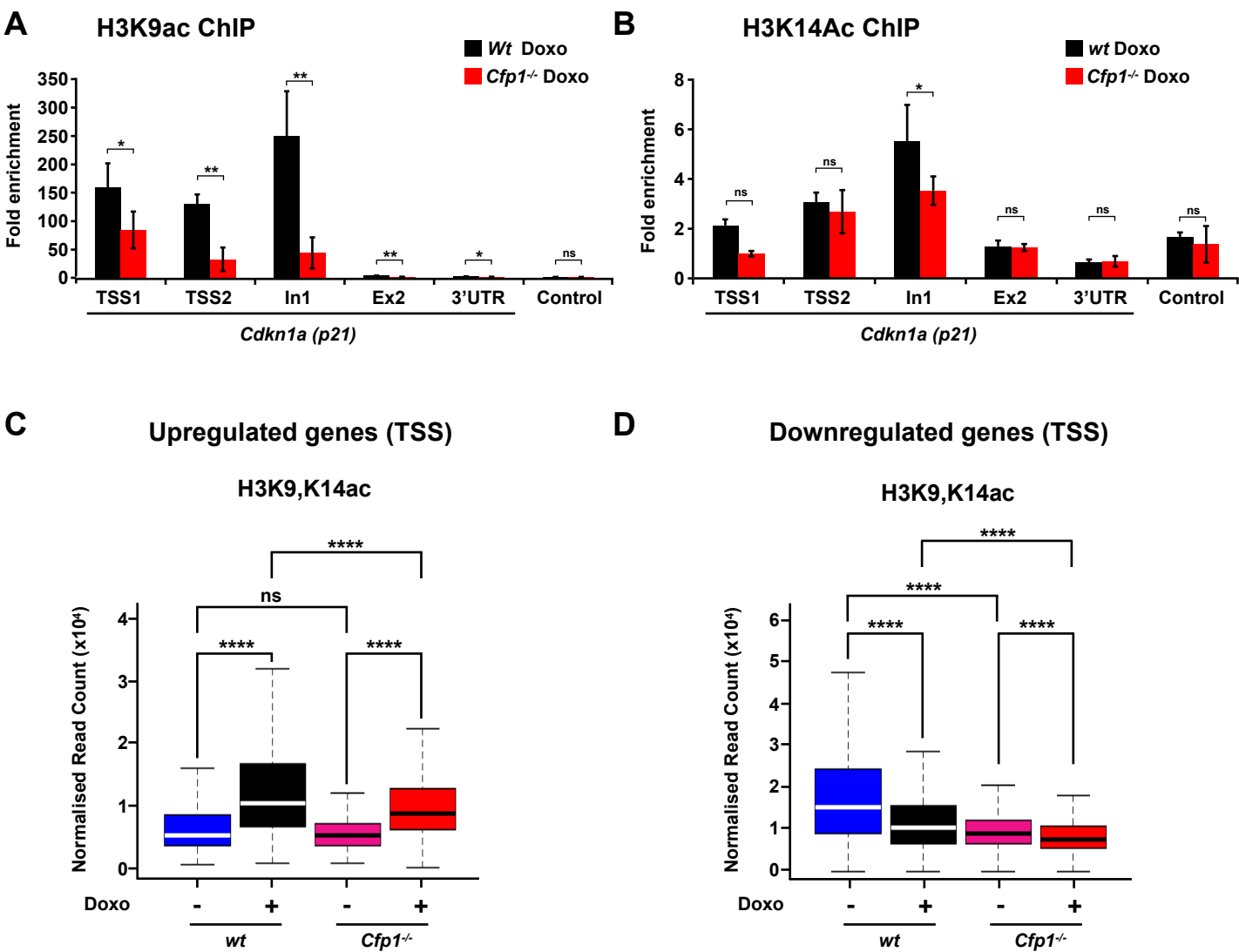

Supplement: Additional file 5: Figure S4. — Cfp1-deficiency associates with decreased H3 acetylation at regulated promoters. [file 13059_2014_451_MOESM5_ESM.pdf]

Clouaire et al. Additional File 7. Figure S5.

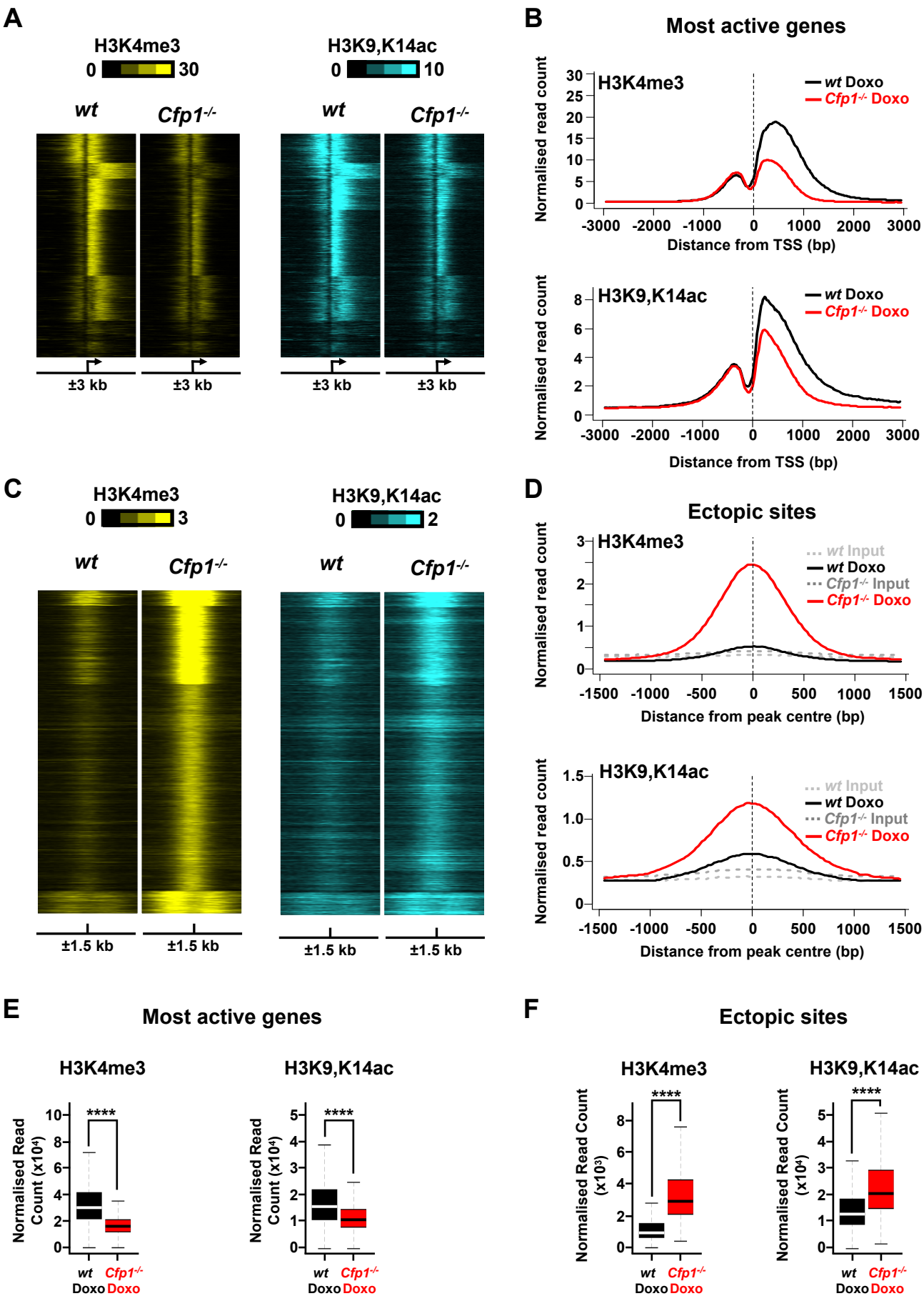

Supplement: Additional file 7: Figure S5. — H3K9 acetylation is altered at Cfp1-regulated H3K4me3 binding sites in doxorubicin-treated cells. [file 13059_2014_451_MOESM7_ESM.pdf]

# Clouaire et al. Additional File 11 Figure S6.

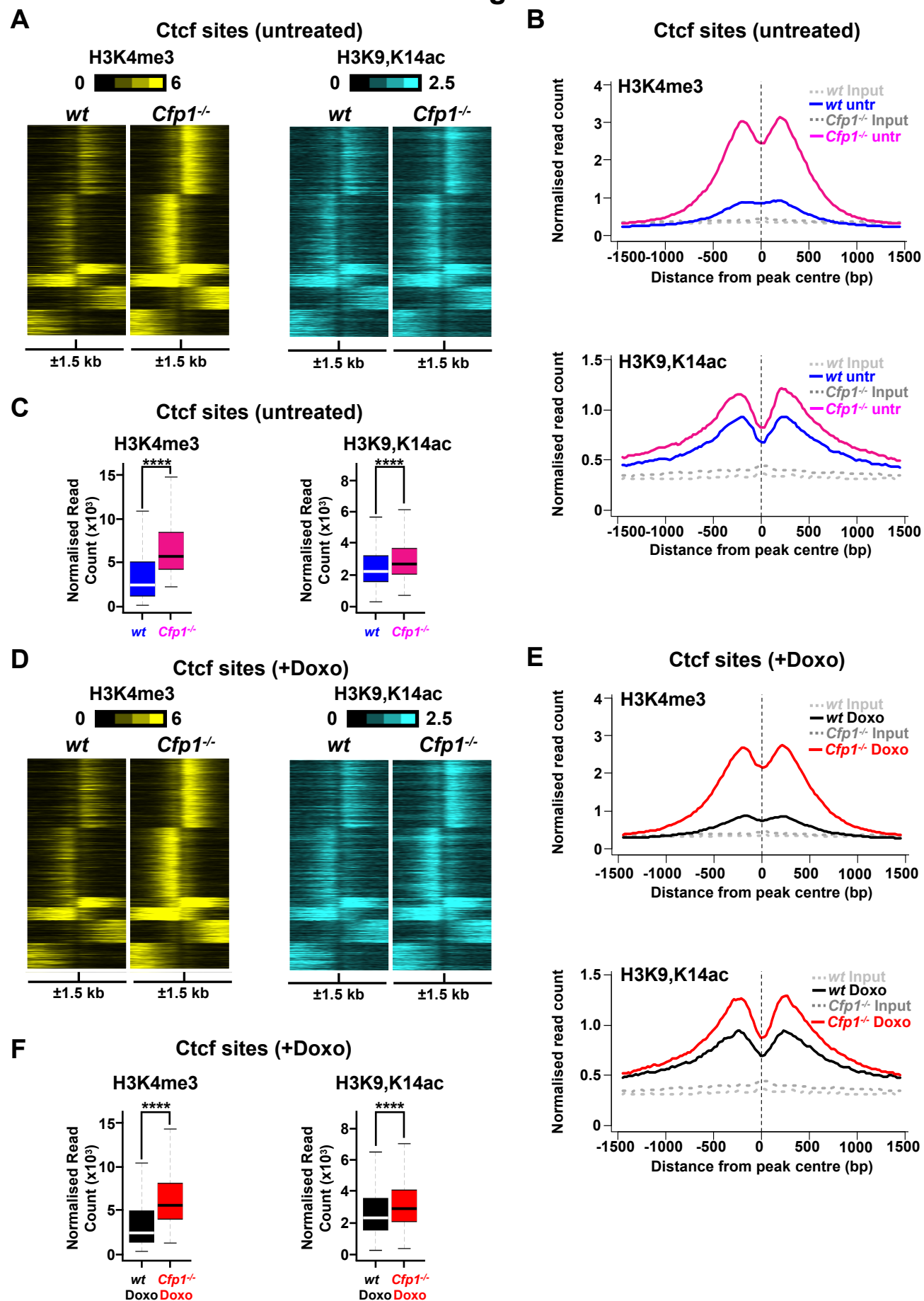

Supplement: Additional file 11: Figure S6. — H3K9 acetylation is altered at Ctcf binding sites in the absence of Cfp1. [file 13059_2014_451_MOESM11_ESM.pdf]

Clouaire et al. Additional File 12 Figure S7.

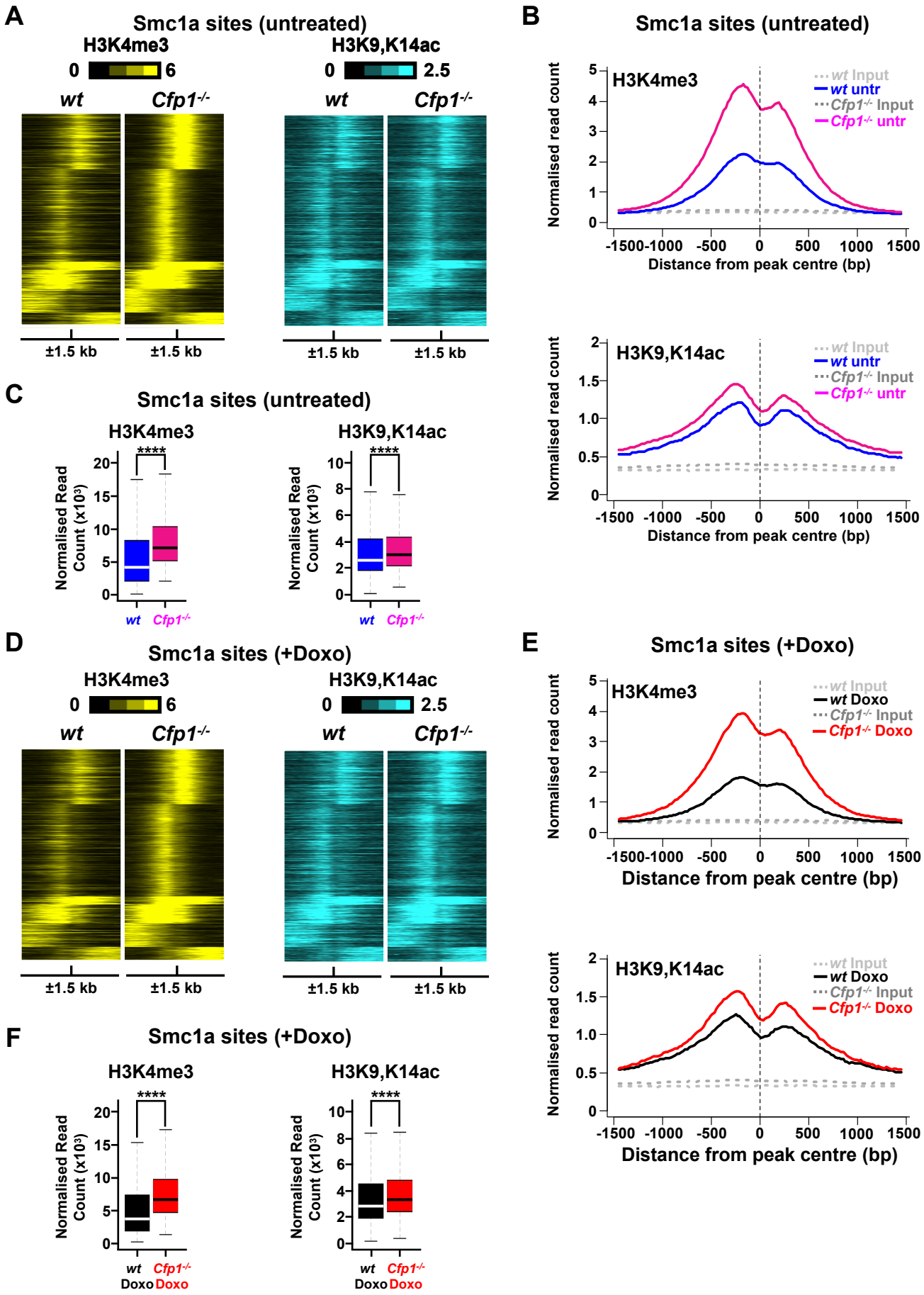

Supplement: Additional file 12: Figure S7. — H3K9 acetylation is altered at cohesin binding sites in the absence of Cfp1. [file 13059_2014_451_MOESM12_ESM.pdf]

Clouaire et al. **Additional File 13 Figure S8.**

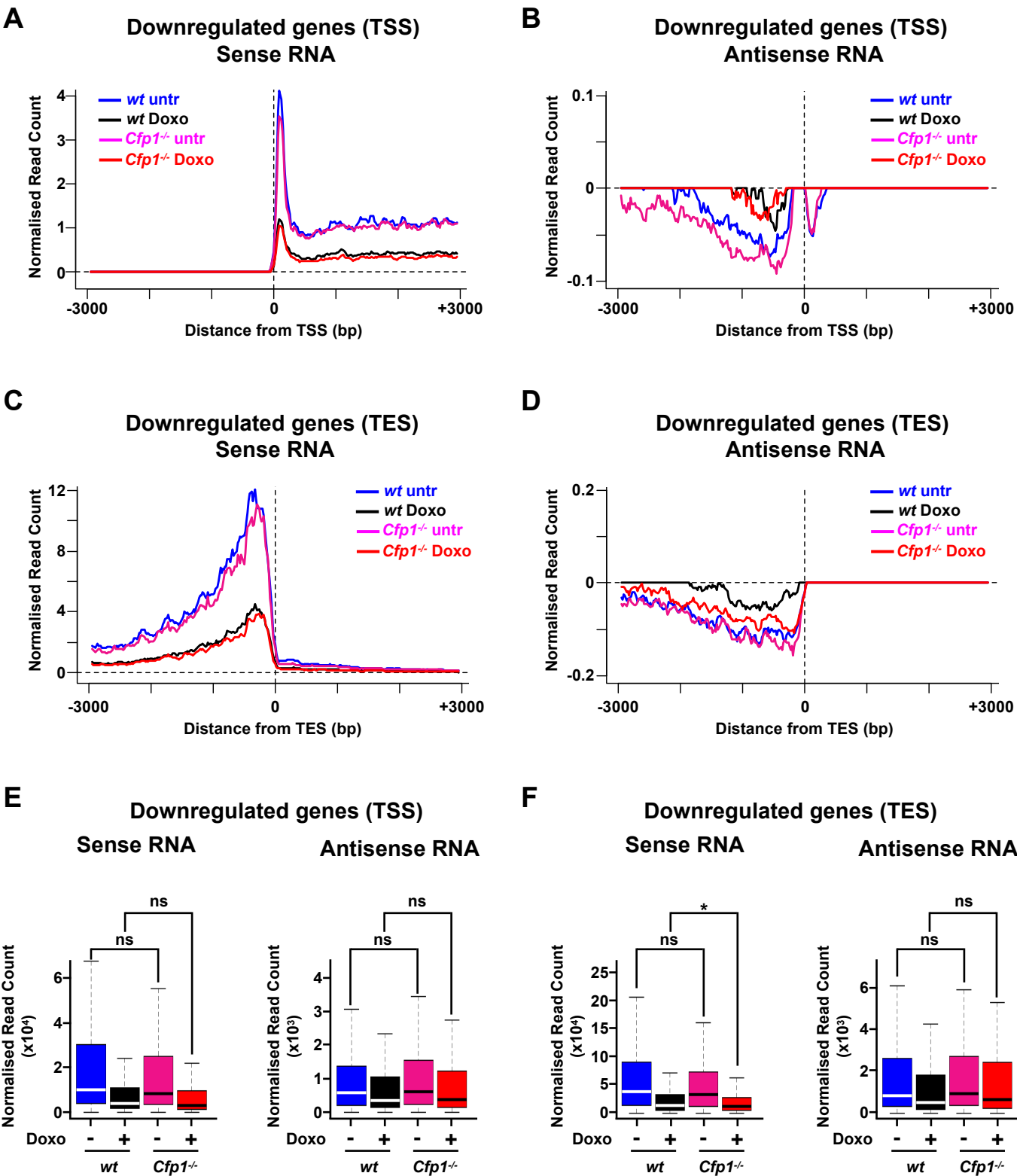

Supplement: Additional file 13: Figure S8. — Transcription at TSSs and TESs of genes downregulated by doxorubicin. [file 13059_2014_451_MOESM13_ESM.pdf]
