# Supplementary material for: Cfp1 is required for gene expression-dependent H3K4 trimethylation and H3K9 acetylation in embryonic stem cells
Source: Genome Biol. 2014 Sep 4;15(9):451. doi: 10.1186/s13059-014-0451-x (PMC4189735; doi:10.1186/s13059-014-0451-x)
Supplement: Additional file 14: Table S6. — List of antibodies used in the study. [file 13059_2014_451_MOESM14_ESM.pdf]

Additional file 14: Table S6. **List of antibodies used in the study**

H3K4me3	Abcam	ab8580.
H3K9,K14ac	Millipore	06-599
H3K27ac	Abcam	ab4729
H3K9ac	Millipore	06-942
H3K14ac	Millipore	07-353
H3	Abcam	ab1791
P53S18P	Cell Signaling	9248L
RNA pol II	Santa Cruz	sc-899
GCN5	Santa Cruz	Sc-20698X
HDAC1	Abcam	ab7028
P53	Santa Cruz	Sc-126
TBP	Abcam	ab28175
