# Supplementary material for: Cfp1 is required for gene expression-dependent H3K4 trimethylation and H3K9 acetylation in embryonic stem cells
Source: Genome Biol. 2014 Sep 4;15(9):451. doi: 10.1186/s13059-014-0451-x (PMC4189735; doi:10.1186/s13059-014-0451-x)
Supplement: Additional file 15: Table S7. — List of oligonucleotides used in the study. [file 13059_2014_451_MOESM15_ESM.pdf]

Additional file 15: Table S7. List of Oligonucleotides used in the study

ChIP

Cdkn1a (p21)	TSS1	F	TGTGACATGTATCAGGTGAAGGA
		R	TGAATGTATAGTCAGGCACACCA
	TSS2	F	GCGCAGATCCACAGCGATA
		R	CGGGCACGCACAAATACAC
	Intron 1	F	GTGGCCCTTGAGAGACAAGGT
		R	GGACTCGGCTAAGGCTGGAT
	Exon 2	F	GTCGCTGTCTTGCACTCTGG
		R	AAGGCCATCCTCAAATGGTG
	3'UTR	F	CCTGGTTCCTTGCCACTTCTT
		R	GCCATCCCTGTTCTAGGCTGT
ActB	TSS	F	TCAGGACCCTGCAGTGAGGT
		R	ACACCCGCCACCAGGTAAG
Gapdh	TSS	F	TTCGCACCAGCATCCCTAGA
		R	TCTTGTGCAGTGCCAGGTGA
Intergenic	chr15	F	GGGACGGGAACAAATGATGA
		R	GACTGCCGTTCTTTGCTTGG
Intergenic	chr5	F	GTGGCTGACACTGTGCTGCT
		R	GGCTGTTTGCCTCCTCTGTC

RTqPCR

Cdkn1a (p21)	F	CCTGGTGATGTCCGACCTGTT
	R	GGGGAATCTTCAGGCCGCTC
Mdm2	F	CAGCTTCGGAACAAGAGACTC
	R	CTGCTCTCACTCAGCGATGT
pou5f1(Oct4)	F	ATGAAAGCCCTGCAGAAGGAG
	R	ACAGATGGTGGTCTGGCTGAA
Nanog	F	AACCAAAGGATGAAGTGCAAGC
	R	TGCAATGGATGCTGGGATACT
Sox2	F	TCGTGGTCTTGTTTAAGGCAAA
	R	AATTACCAACGATATCAACCTGCAT
ActB	F	TGACGGCCAGGTCATCACT
	R	AGTTTCATGGATGCCACAGGA
Gapdh	F	GCCATGTAGGCCATGAGGTC
	R	CCAGGTTGTCTCCTGCGACT
Lmna (LaminA)	F	GCTTCCCACCGAAGTTCACC
	R	GTGTTCTGCGCCTTCCACAC
ERCC-00130	F	ACGGGACAAGGGATCAACCT
	R	GGCGAATGACAGTGGAAAGC
ERCC-00096	F	CGCAGACGGTATCAACAGGA
	R	TGGACACTGCATCGGAAGAC
ERCC-00074	F	GCTTCCCATCTTCTTTGAGAGTTG
	R	CCAATTGGAGCTTTCTTAGCTGTC

Splicing

Splice 1	F	GTGAGGAGGAGCATGAATGG
	R	CGAAGAGACAACGGCACACTTT
Splice 2	F	GTGTCAGAGTCTAGGGGAATTGGA
	R	GAACAGGTCGGACATCACCAG
Splice 3	F	GCCTTGTCGCTGTCTTGCAC
	R	AAGAGGCCTCCTGACCCACA
Unspliced	F	GTGGCCCTTGAGAGACAAGGT
	R	GGACTCGGCTAAGGCTGGAT
Total RNA	F	CCTGGTTCCTTGCCACTTCTT
	R	GCCATCCCTGTTCTAGGCTGT
